# Psychometric evidence of a perception scale about covid-19 vaccination process in Peruvian dentists: a preliminary validation

**DOI:** 10.1186/s12913-022-08677-w

**Published:** 2022-10-28

**Authors:** César F. Cayo-Rojas, Nancy Córdova-Limaylla, Gissela Briceño-Vergel, Marysela Ladera-Castañeda, Hernán Cachay-Criado, Carlos López-Gurreonero, Alberto Cornejo-Pinto, Luis Cervantes-Ganoza

**Affiliations:** 1 Universidad Privada San Juan Bautista, School of Stomatology, Av. Jose Antonio Lavalle Avenue s/n (Ex Hacienda Villa); Chorrillos, Lima, Peru; 2 Universidad Nacional Federico Villarreal, Postgraduate School, “Grupo de Investigación Salud y Bienestar Global”, Lima, Peru; 3 Universidad Científica del Sur, School of Stomatology, Lima, Peru; 4 Universidad Inca Garcilaso de la Vega, Faculty of Stomatology, Lima, Peru

**Keywords:** COVID-19, Dentistry, Dentists, Perception, Peru, Vaccination

## Abstract

**Background:**

In the current pandemic context, dental professionals have greater occupational risks due to their healthcare activity, placing their expectations on the vaccine as a means of protection and at the same time hoping that the immunization process will be safe, reliable and comfortable, giving them greater peace of mind when they return to work. Therefore, the aim of the present study was to develop and provide a preliminary validation of a scale to measure perception of the COVID-19 vaccination process in Peruvian dental professionals.

**Methods:**

Cross-sectional study with instrumental design. The scale was self-administered virtually. It was distributed through social networks to 220 dental professionals from two universities in the Peruvian capital between June and August 2021. The Aiken V was used for content analysis, while descriptive statistics such as mean, variance, kurtosis and skewness were used for construct validation, in addition to Pearson’s correlation matrix for analysis of the 18 items. Subsequently, a Parallel Analysis based on minimum rank factor analysis was performed. Finally, the reliability of the total scale and its dimensions was evaluated with Cronbach’s alpha.

**Results:**

The Aiken V coefficient values were favorable for all items. Parallel analysis indicated the existence of three dimensions. Principal component analysis with rotation suggested grouping eight items for the first dimension, six items for the second dimension and four items for the third dimension. These dimensions showed good reliability, as Cronbach’s alpha was 0.87, (95% confidence interval [CI]: 0.84–0.90), 0.80 (95% CI: 0.75–0.84) and 0.82 (95% CI: 0.78–0.86), respectively. In addition, the overall reliability of the scale was 0.89 (95% CI: 0.86–0.91), being acceptable.

**Conclusions:**

The perception scale of the COVID-19 vaccination process in dental professionals proved preliminarily to be a valid and reliable scale that can be used for research purposes. However, it is recommended to extend its application and evaluate its metric properties in other health professionals.

## Background

An unexpected event such as the COVID-19 pandemic, which suddenly interrupted the “normality” of daily activities, has generated various effects on physical and mental health of the population due to uncertainty about various problems, including preventive measures to contain the spread, morbidity and mortality of the disease [1, 2].

In this context, according to the Occupational Safety and Health Administration [3] dental professionals are in the very high risk category for exposure to SARS-CoV-2 virus during care procedures that generate contaminated bioaerosols [3–5]. The vaccine seems to be a hopeful strategy to reestablish normality, and the scope it may have depends on perception and acceptance of the immunization process established under the guidelines of each responsible entity, since its successful implementation would lead to greater receptivity and safety of general public, considering that there will be future calls for this purpose.

Another point to consider is infodemics on social networks and media about effectiveness of the vaccine [6–8], and if the vaccination process is carried out in a disorganized manner, it would result in low levels of acceptance. Therefore, it is important to pay attention to administrative management, such as the dissemination of the list of those selected for vaccination, date, schedule, geographical location of the vaccination station, the conditions of the vaccination environment and the vaccinators. It is also important to take into account the perception of service by valuing the care provided by the staff in charge, the perception of healthcare procedures through the performance of health team, and to provide adequate information about benefits of the vaccine, as well as the occurrence of possible adverse reactions [9].

In Peru, the case fatality rate due to the second wave was 9.32% [10], the highest in the world, with an accumulated number of infections and deaths of 2,148,418 and 198,167 respectively up to August, making it even more important to have a vaccination process that guarantees good acceptance by health professionals and public in general.

To date, several studies have been carried out to assess the acceptance level of the vaccine [6, 11–17] such as the ICPCovid questionnaire that collects data on vaccine acceptability at different levels of effectiveness, perceptions of COVID-19 vaccination, knowledge about the vaccine and vaccination in the population (Jaramillo-Monge). In the study by Jaramillo-Monge et al. it was found that 91% of participants were willing to be vaccinated with a COVID-19 Vaccine if it has at least 95% efficacy, 68.5% if it has at least 90% efficacy and 40.5% if it has at least 70% efficacy. Approximately 55.5% of the participants indicated that they feared unexpected side effects. Likewise, the study by Alvarado-Socarras et al. reported that between 77 and 90.7% would accept COVID-19 vaccination with a vaccine efficacy of 60 and 80%, respectively. On the other hand, Kabamba et al. indicated that only 28% of participants would agree to receive a COVID-19 vaccine when one became available. However, although these results show vaccine acceptance, no research has yet included the perception regarding immunization process, which is of vital importance for the relevant authorities in order to rethink their administrative and operational strategies in the search for greater receptivity and safety of public in general. Therefore, the aim of the present study was to develop and provide a preliminary validation of a scale to measure perception of the COVID-19 vaccination process in Peruvian dental professionals.

## Methods

### Bioethical considerations

The present study respected the bioethical principles for medical research on human beings of the Declaration of Helsinki [18], related to confidentiality, freedom, respect and non-maleficence; and, was approved by the Institutional Research Ethics Committee of the Universidad Privada San Juan Bautista with resolution No. 423–2021-CIEI-UPSJB dated July 1, 2021.

### Type of study

An analytical, observational, cross-sectional, prospective study with an instrumental design was conducted. This manuscript was written according to the STrengthening the Reporting of OBservational studies in Epidemiology (STROBE) guidelines for observational studies [19].

### Population and selection of participants

**Fig. 1:**
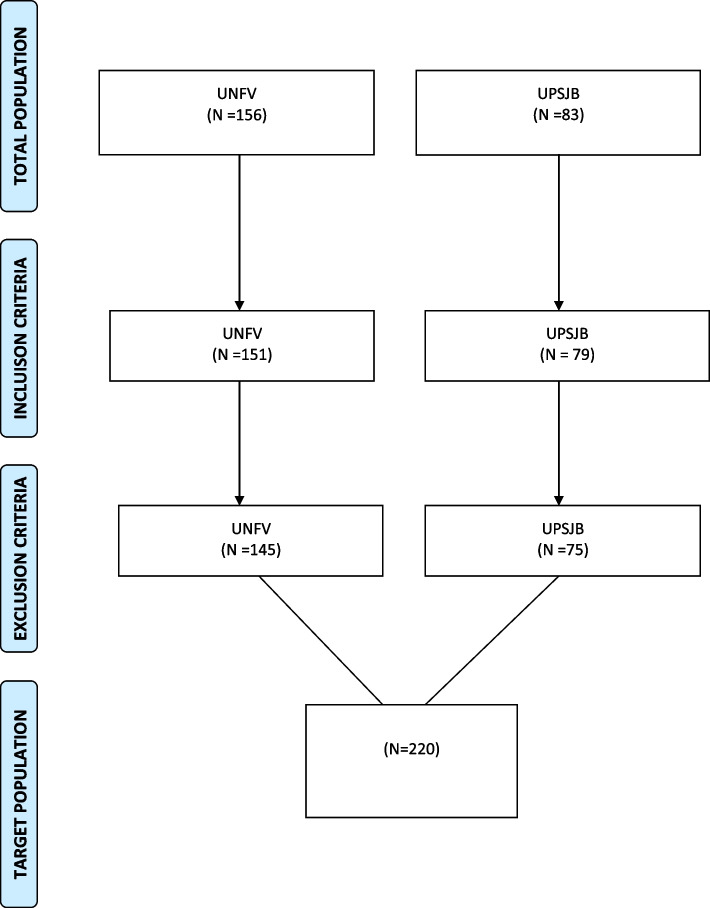
Selection of target population according to eligibility criteria

The study was carried out between July and August 2021 at the Universidad Nacional Federico Villarreal (UNFV) and the Universidad Privada San Juan Bautista (UPSJB), Lima, Peru. The initial population consisted of 239 professional dentists, 156 from the UNFV and 83 from the UPSJB (in both universities, including professors and graduate students). It was not necessary to calculate a sample size, since the entire population was included according to the selection criteria. This allowed us to have a target population of 220 participants. (Fig. 1).

#### Inclusion criteria

- Professionals with general dentistry degree.
- Dentists affiliated to a professional association.
- Dentists who have been administered the vaccine for COVID-19.
- Dentists who gave virtual informed consent.

#### Exclusion criteria

- Dentists who did not complete the virtual questionnaire.

### Preparation of instrument

A review of the literature available in Pubmed and Scopus databases was carried out in order to construct the theoretical framework and conceptually define the construct. Next, a scale-type documentary instrument was developed and underwent the process of validation of content, construct and reliability.

### Procedure

- The P-Vac-Cov19-DENT scale was designed, analyzed and reviewed by the researchers.
- The questionnaire items were created from the literature and from key words previously noted from an in-depth interview conducted with 50 dentists. This was conducted by three experts in dental public health research who designed a scale with four dimensions aimed at assessing the perception of the vaccination process organization, the perception of the vaccine efficacy, the general perception of the service and the perception of the care procedure. Next, a focused interview (focus group) was conducted with 20 experiential judges (undergraduate and postgraduate professors and postgraduate students) in order to identify any lack of clarity in the items or to identify ambiguous questions.
- Subsequently, the items of the four dimensions were validated through the judgment of five experts with more than 15 years of experience (two researchers with a Doctor of Public Health degree, a researcher specializing in public health with a Doctor of Dentistry degree, a specialist in biostatistics and a Master in Stomatology) who reviewed the clarity, objectivity, timeliness, organization, sufficiency, intentionality, consistency, coherence and methodology of instrument.
- The P-Vac-Cov19-DENT scale consisted of 18 items on perception of the COVID-19 vaccination process. Each item had five ordinal (Likert-type) response alternatives: “strongly disagree”, “disagree”, “neutral”, “agree” and “strongly agree”, with scores from 1 to 5 respectively.
- The P-Vac-Cov19-DENT scale was transferred to the Google Classroom (Mountain View, California, USA) (available between July 1 and August 15, 2021) and was distributed in a self-administered manner through a link via WhatsApp (Facebook Inc., Menlo Park, California, USA and Facebook (White Plains, NY, USA) social networks, as well as institutional e-mails. The invitation was extended to professional dentists from UNFV and UPSJB. Upon clicking on the invitation, participants were automatically directed to the objective of study and to the informed consent page with contact information of the principal investigator. Once they agreed to participate, they were directed to the scale with its indications to develop it. However, they were free to decline the assessment if during its development they did not wish to complete it. Personal details such as telephone number, name and address were not required. The study was designed so that they could respond only once. The data were collected and stored in an Excel 2019 spreadsheet (Microsoft, Redmond, Washington, USA) which was stored in a digital folder with password access only for the researchers.

### Statistical analysis

For content validity, all items were evaluated by five expert judges with Likert scale scores from 1 (strongly disagree) to 5 (strongly agree). These scores were used to calculate the Aiken V coefficient with its 95% confidence interval [20] according to the criteria of clarity, objectivity, timeliness, organization, sufficiency, intentionality, consistency, coherence and methodology.

For construct validation, a descriptive analysis was performed to calculate the mean, variance, skewness and kurtosis of the scale items. The value taken into account for skewness was ±1.5 [21]. In addition, the variability of each item was assessed, verifying with Pearson’s correlation matrix (item - total) the negative items and then proceeding to invert the Likert scale scores. An Exploratory Factor Analysis (EFA) was then performed on the instrument, considering the coefficient of determination (< 0.05), a Kaiser-Meier-Olkin measure (KMO > 0.5) and Bartlett’s sphericity (*p* < 0.05) as acceptable. The number of dimensions of P-Vac-Cov19-DENT scale was determined according to principal component analysis, taking into consideration the variance explained with respect to the total variance. Then, to group the items (Q) according to the dimensions, Principal Component Analysis (PCA) was performed with the Varimax rotation method with Kaiser normalization. In addition, Parallel Analysis based on minimum rank factor analysis was performed to extract the factors appropriately (Factor Analysis v12.01.01, Universitat Rovira i Virgili, Spain). Subsequently, the reliability of the total scale and each of its dimensions was analyzed with Cronbach’s alpha, considering it acceptable (≥0.75).

The data analysis was imported and performed with the SPSS statistical package (Statistical Package for the Social Sciences, IBM Corp., Armonk, NY, USA) version 24.0.

## Results

The majority of surveyed dentists were between 41 and 65 years of age (53.18%), being predominantly female with 59.55% of the total. 51.36% were married and 38.18% had no children. In addition, 70.45% of the dentists were from the capital city and 54.09% were only in private practice. The majority (65.45%) had 10 or more years of experience and 54.55% of the total had a master’s degree. Of these, 55.91% did not have a specialty. On the other hand, 79.09% did not report vulnerability to COVID-19 and 76.36% did not report a history of COVID-19. Finally, 85% received the Sinopharm vaccine and 86.82% reported having received two doses (Table 1).

**Table 1:** Socio-demographic characteristics of dental professionals

Variable	Categories	Frequency	Percentage
**Age**	< 41 years	97	44.09
	41–65 years	117	53.18
	> 65 years	6	2.73
**Sex**	Female	131	59.55
	Male	89	40.45
**Marital status**	Single	88	40.00
	Married (living alone)	13	5.91
	Casado (living with family)	100	45.45
	Widower	2	0.91
	Divorced	17	7.73
**Children**	No children	84	38.18
	One child	57	25.91
	Two children	67	30.45
	Three children or more	12	5.45
**Origin**	Capital	155	70.45
	Province	65	29.55
**Occupation**	Private care	119	54.09
	Public care	27	12.27
	University professor	21	9.55
	Teaching and private care	35	15.91
	Teaching and public care	18	8.18
**Years of experience**	Less than 10 years	76	34.55
	10 years or more	144	65.45
**Academic degree**	Bachelor’s degree	88	40.00
	Master’s degree	120	54.55
	Doctorate	12	5.45
**Specialization degree**	Yes	97	44.09
	No	123	55.91
**Vulnerability to COVID-19**	Yes	46	20.91
	No	174	79.09
**History of COVID-19**	Yes	52	23.64
	No	168	76.36
**Vaccine**	Sinopharm	187	85.00
	Pfizer/BioNtech	26	11.82
	Other	7	3.18
**Dose**	1 dose	26	11.82
	2 doses	191	86.82
	More than 2 doses	3	1.36

According to Aiken’s V coefficient, from the scores obtained by the five expert judges, it was obtained that the eighteen items showed values ranging from 0.85 to 1.00 (V ≥ 0.70) for the criteria of clarity, objectivity, timeliness, organization, sufficiency, intentionality, consistency, coherence and methodology, this being very acceptable. In addition, the lower limit of all 95% confidence intervals were acceptable (Table 2).

**Table 2:** Aiken’s V for the evaluation of the clarity, objectivity, timeliness, organization, sufficiency, intentionality, consistency, coherence and methodology of the P-Vac-Cov19-DENT scale items

Item	Clarity		Objectivity		Timeliness		Organization		Sufficiency		Intentionality		Consistency		Coherence		Methodology	
	V	95% CI	V	95% CI	V	95% CI	V	95% CI	V	95% CI	V	95% CI	V	95% CI	V	95% CI	V	95% CI
**Q1**	0.95	0.76–0.99	0.90	0.70–0.97	0.90	0.70–0.97	0.90	0.70–0.97	0.90	0.70–0.97	0.95	0.76–0.99	0.90	0.70–0.97	0.95	0.76–0.99	0.95	0.76–0.99
**Q2**	0.95	0.76–0.99	0.95	0.76–0.99	0.95	0.76–0.99	0.95	0.76–0.99	0.90	0.70–0.97	0.95	0.76–0.99	0.90	0.70–0.97	0.95	0.76–0.99	0.95	0.76–0.99
**Q3**	1.00	0.84–1.00	0.95	0.76–0.99	0.95	0.76–0.99	0.90	0.70–0.97	0.90	0.70–0.97	1.00	0.84–1.00	0.95	0.76–0.99	0.95	0.76–0.99	0.95	0.76–0.99
**Q4**	0.95	0.76–0.99	0.95	0.76–0.99	0.90	0.70–0.97	0.95	0.76–0.99	0.90	0.70–0.97	0.90	0.70–0.97	0.95	0.76–0.99	0.90	0.70–0.97	0.90	0.70–0.97
**Q5**	0.95	0.76–0.99	0.95	0.76–0.99	0.90	0.70–0.97	0.95	0.76–0.99	0.90	0.70–0.97	0.95	0.76–0.99	0.90	0.70–0.97	0.95	0.76–0.99	0.95	0.76–0.99
**Q6**	0.95	0.76–0.99	0.95	0.76–0.99	0.95	0.76–0.99	0.95	0.76–0.99	0.90	0.70–0.97	0.95	0.76–0.99	0.90	0.70–0.97	0.90	0.70–0.97	0.90	0.70–0.97
**Q7**	0.90	0.70–0.97	0.90	0.70–0.97	0.95	0.76–0.99	0.90	0.70–0.97	0.95	0.76–0.99	0.90	0.70–0.97	0.95	0.76–0.99	0.90	0.70–0.97	0.95	0.76–0.99
**Q8**	0.95	0.76–0.99	0.95	0.76–0.99	0.95	0.76–0.99	0.95	0.76–0.99	0.95	0.76–0.99	1.00	0.84–1.00	0.95	0.76–0.99	0.95	0.76–0.99	0.95	0.76–0.99
**Q9**	1.00	0.84–1.00	1.00	0.84–1.00	1.00	0.84–1.00	0.95	0.76–0.99	0.95	0.76–0.99	1.00	0.84–1.00	1.00	0.84–1.00	1.00	0.84–1.00	0.90	0.70–0.97
**Q10**	0.90	0.70–0.97	0.90	0.70–0.97	0.90	0.70–0.97	0.90	0.70–0.97	1.00	0.84–1.00	0.90	0.70–0.97	0.95	0.76–0.99	0.90	0.70–0.97	0.90	0.70–0.97
**Q11**	0.95	0.76–0.99	0.95	0.76–0.99	0.95	0.76–0.99	0.95	0.76–0.99	0.90	0.70–0.97	0.95	0.76–0.99	0.90	0.70–0.97	0.90	0.70–0.97	0.90	0.70–0.97
**Q12**	0.95	0.76–0.99	0.90	0.70–0.97	0.90	0.70–0.97	0.95	0.76–0.99	0.90	0.70–0.97	0.95	0.76–0.99	0.90	0.70–0.97	0.90	0.70–0.97	0.90	0.70–0.97
**Q13**	0.95	0.76–0.99	0.95	0.76–0.99	0.95	0.76–0.99	0.95	0.76–0.99	1.00	0.84–1.00	0.95	0.76–0.99	0.95	0.76–0.99	0.95	0.76–0.99	1.00	0.84–1.00
**Q14**	0.90	0.70–0.97	0.90	0.70–0.97	0.95	0.76–0.99	0.95	0.76–0.99	0.95	0.76–0.99	0.95	0.76–0.99	0.90	0.70–0.97	0.90	0.70–0.97	0.90	0.70–0.97
**Q15**	1.00	0.84–1.00	1.00	0.84–1.00	0.95	0.76–0.99	0.95	0.76–0.99	0.95	0.76–0.99	1.00	0.84–1.00	1.00	0.84–1.00	0.90	0.70–0.97	0.95	0.76–0.99
**Q16**	0.90	0.70–0.97	0.90	0.70–0.97	0.90	0.70–0.97	0.95	0.76–0.99	0.90	0.70–0.97	0.90	0.70–0.97	0.90	0.70–0.97	0.90	0.70–0.97	0.90	0.70–0.97
**Q17**	1.00	0.84–1.00	0.90	0.70–0.97	0.90	0.70–0.97	1.00	0.84–1.00	0.90	0.70–0.97	0.90	0.70–0.97	0.90	0.70–0.97	0.90	0.70–0.97	0.90	0.70–0.97
**Q18**	0.90	0.70–0.97	0.90	0.70–0.97	0.90	0.70–0.97	0.95	0.76–0.99	0.95	0.76–0.99	0.90	0.70–0.97	0.90	0.70–0.97	0.90	0.70–0.97	0.90	0.70–0.97

Aiken’s V coefficient, 95% CI

According to descriptive analysis of the P-Vac-Cov19-DENT scale, it was noted that item 6 obtained the highest mean score with 4.14, while item Q15 obtained the lowest mean score with 2.40. Regarding response variability, item Q1 obtained the highest variance with 1.73, while item Q11 obtained the lowest variance with 0.64. Asymmetry values of the scale items did not exceed the acceptable range (± 1.5) [21] (Table 3).

**Table 3:** Descriptives of the P-Vac-Cov19-DENT scale

Item	Mean	Variance	Skewness	Kurtosis
**Q1**	3.21	1.73	−0.28	−1.25
**Q2**	3.78	1.22	−1.12	0.53
**Q3**	3.12	1.64	−0.26	−1.25
**Q4**	4.06	0.69	−1.09	1.60
**Q5**	3.85	1.08	−1.03	0.59
**Q6**	4.14	0.72	−1.23	2.29
**Q7**	3.23	1.83	−0.34	−1.23
**Q8**	3.54	1.30	−0.63	− 0.59
**Q9**	3.62	1.18	−0.90	0.13
**Q10**	2.92	1.74	−0.06	−1.36
**Q11**	4.00	0.64	−1.19	2.43
**Q12**	4.04	0.77	−1.31	2.16
**Q13**	2.60	1.35	0.42	−0.89
**Q14**	3.47	1.16	−0.48	− 0.57
**Q15**	2.40	0.96	0.61	−0.19
**Q16**	3.68	1.12	−0.83	−0.04
**Q17**	3.53	1.32	−0.48	−0.87
**Q18**	3.43	1.37	−0.38	−0.98
**Total**	62.62	107.67	−0.09	−0.08

On analyzing the item-total correlation and covariance of the P-Vac-Cov19-DENT scale, two items were found to be negatively correlated with respect to the total score. Therefore, we proceeded to invert the evaluation of responses according to the Likert scale, with 1 being strongly agree and 5 being strongly disagree. Finally, with this adjustment it was obtained that all the items correlated positively and significantly (> 0.35) with the total score (Table 4).

**Table 4:** Identification of negative and positive reagents, according to item-total analysis of the P-Vac-Cov19-DENT scale

Item	Initial		Final	
	Item-total correlation^**a**^	Covariance	Item-total correlation^**a**^	Covariance
**Q1**	0.70	9.52	0.68	10.29
**Q2**	0.66	7.61	0.66	8.43
**Q3**	0.68	9.06	0.68	9.97
**Q4**	0.57	4.92	0.63	6.07
**Q5**	0.57	6.12	0.60	7.19
**Q6**	0.62	5.41	0.62	6.05
**Q7**	0.70	9.79	0.68	10.68
**Q8**	0.58	6.91	0.56	7.42
**Q9**	0.71	7.98	0.70	8.77
**Q10**	0.74	10.08	0.69	10.47
**Q11**	0.60	4.98	0.65	6.03
**Q12**	0.54	4.93	0.55	5.59
**Q13**	−0.10	−1.21	0.36	4.82
**Q14**	0.33	3.69	0.51	6.31
**Q15**	−0.18	−1.87	0.42	4.72
**Q16**	0.56	6.17	0.55	6.75
**Q17**	0.55	6.51	0.49	6.50
**Q18**	0.58	7.06	0.51	6.85

^**a**^Based on Pearson’s correlation

When analyzing the inter-item correlation matrix of the P-Vac-Cov19-DENT scale, it was observed that the determinant value was significant (*p* = 0.000). This being one of the requirements to perform the exploratory factor analysis (Table 5).

**Table 5:** Inter-item correlation matrix of P-Vac-Cov19-DENT scale

Item	Q1	Q2	Q3	Q4	Q5	Q6	Q7	Q8	Q9	Q10	Q11	Q12	Q13i	Q14	Q15i	Q16	Q17	Q18
**Q1**	1.00	0.47	0.56	0.35	0.35	0.33	0.58	0.40	0.56	0.65	0.30	0.24	0.15	0.16	0.12	0.25	0.18	0.22
**Q2**	0.47	1.00	0.52	0.45	0.43	0.42	0.44	0.45	0.51	0.46	0.46	0.31	0.18	0.22	0.17	0.21	0.15	0.14
**Q3**	0.56	0.52	1.00	0.32	0.39	0.31	0.45	0.36	0.48	0.52	0.34	0.30	0.15	0.22	0.18	0.29	0.23	0.31
**Q4**	0.35	0.45	0.32	1.00	0.54	0.49	0.33	0.28	0.39	0.30	0.67	0.25	0.26	0.42	0.40	0.22	0.18	0.18
**Q5**	0.35	0.43	0.39	0.54	1.00	0.50	0.30	0.18	0.34	0.39	0.52	0.28	0.26	0.28	0.23	0.18	0.22	0.16
**Q6**	0.33	0.42	0.31	0.49	0.50	1.00	0.37	0.30	0.42	0.32	0.45	0.33	0.16	0.26	0.20	0.30	0.34	0.31
**Q7**	0.58	0.44	0.45	0.33	0.30	0.37	1.00	0.47	0.52	0.58	0.39	0.27	0.14	0.27	0.17	0.24	0.22	0.23
**Q8**	0.40	0.45	0.36	0.28	0.18	0.30	0.47	1.00	0.56	0.39	0.27	0.20	0.09	0.19	0.12	0.16	0.17	0.25
**Q9**	0.56	0.51	0.48	0.39	0.34	0.42	0.52	0.56	1.00	0.54	0.41	0.19	0.21	0.15	0.12	0.27	0.25	0.34
**Q10**	0.65	0.46	0.52	0.30	0.39	0.32	0.58	0.39	0.54	1.00	0.36	0.29	0.05	0.15	0.09	0.34	0.27	0.28
**Q11**	0.30	0.46	0.34	0.67	0.52	0.45	0.39	0.27	0.41	0.36	1.00	0.33	0.23	0.42	0.40	0.26	0.20	0.24
**Q12**	0.24	0.31	0.30	0.25	0.28	0.33	0.27	0.20	0.19	0.29	0.33	1.00	0.10	0.38	0.27	0.56	0.35	0.39
**Q13i**	0.15	0.18	0.15	0.26	0.26	0.16	0.14	0.09	0.21	0.05	0.23	0.10	1.00	0.48	0.40	0.11	−0.03	−0.06
**Q14**	0.16	0.22	0.22	0.42	0.28	0.26	0.27	0.19	0.15	0.15	0.42	0.38	0.48	1.00	0.66	0.23	0.07	0.03
**Q15i**	0.12	0.17	0.18	0.40	0.23	0.20	0.17	0.12	0.12	0.09	0.40	0.27	0.40	0.66	1.00	0.15	0.02	−0.01
**Q16**	0.25	0.21	0.29	0.22	0.18	0.30	0.24	0.16	0.27	0.34	0.26	0.56	0.11	0.23	0.15	1.00	0.57	0.56
**Q17**	0.18	0.15	0.23	0.18	0.22	0.34	0.22	0.17	0.25	0.27	0.20	0.35	−0.03	0.07	0.02	0.57	1.00	0.76
**Q18**	0.22	0.14	0.31	0.18	0.16	0.31	0.23	0.25	0.34	0.28	0.24	0.39	−0.06	0.03	−0.01	0.56	0.76	1.00

Determinant = 0.000

The pertinence of the exploratory factor analysis is justified by the sample adequacy indices. The Kaiser-Meyer-Olkin value (KMO = 0.864) and Bartlett’s test of sphericity (1847.93; gl = 153; p = 0.000) were very good. In addition, according to the sum of squared loadings extraction method, a four-dimensional structural model with eigenvalues greater than 1 was obtained, where the four factors obtained explain 64.156% of total variance of the test. However, according to the parallel analysis, the eighteen items of the P-Vac-Cov19-DENT scale were saturated in three factors that explained 62.82% of the total variance of the analysis (Table 6).

**Table 6:** Exploratory factor analysis of P-Vac-Cov19-DENT scale

Item	Total variance explained (%)					
	Initial eigenvalues			Parallel Analysis ^**a**^		
	Total	Variance	Accumulated	Real-data	Mean of random	95th percentile of random
**Q1**	6.40	35.55	35.55	39.29	11.44	12.72
**Q2**	2.19	12.19	47.74	12.74	10.44	11.49
**Q3**	1.84	10.23	57.97	10.80	9.63	10.57
**Q4**	1.11	6.19	64.16	6.42	8.93	9.70
**Q5**	0.84	4.69	68.85	5.01	8.27	8.97
**Q6**	0.78	4.32	73.16	4.07	7.62	8.22
**Q7**	0.66	3.69	76.85	3.73	7.01	7.52
**Q8**	0.61	3.38	80.23	3.14	6.41	6.90
**Q9**	0.52	2.91	83.13	2.65	5.84	6.35
**Q10**	0.45	2.52	85.65	2.58	5.21	5.78
**Q11**	0.44	2.43	88.09	2.17	4.60	5.23
**Q12**	0.39	2.17	90.26	2.04	4.00	4.63
**Q13i**	0.38	2.11	92.37	1.87	3.39	4.09
**Q14**	0.34	1.91	94.28	1.61	2.77	3.49
**Q15i**	0.30	1.69	95.97	1.36	2.13	2.86
**Q16**	0.29	1.59	97.56	0.41	1.50	2.22
**Q17**	0.26	1.42	98.98	0.10	0.81	1.51
**Q18**	0.18	1.02	100.00			

^a^Parallel Analysis based on minimum rank factor analysis

Advised number of dimensions: 3

The factor analysis according to principal component analysis extraction method with Varimax rotation and Kaiser normalization grouped items Q1, Q2, Q3, Q6, Q7, Q8, Q9 and Q10 for the first dimension, with factor loadings ranging from 0.32 to 0.86. For the second dimension, items Q4, Q5, Q11, Q13i, Q14 and Q15i were grouped together, with factor loadings ranging from 0.34 to 0.87. Finally, for the third dimension, items Q12, Q16, Q17 and Q18 were grouped together, with factor loadings ranging from 0.47 to 0.89 (Table 7).

**Table 7:** Principal component analysis of the P-Vac-Cov19-DENT scale with varimax rotation

Item	Rotated component matrix		
	Dimensions		
	Factor 1	Factor 2	Factor 3
**Q1**	0.86		
**Q2**	0.69		
**Q3**	0.64		
**Q4**		0.52	
**Q5**		0.34	
**Q6**	0.32		
**Q7**	0.72		
**Q8**	0.62		
**Q9**	0.80		
**Q10**	0.80		
**Q11**		0.51	
**Q12**			0.47
**Q13**		0.55	
**Q14**		0.87	
**Q15**		0.82	
**Q16**			0.71
**Q17**			0.89
**Q18**			0.89

^a^Loadings lower than absolute 0.300 omitted

According to the grouping of items after performing the rotated loading matrix, Cronbach’s reliability analysis was performed for the first (0.87, 95% CI: 0.84–0.90), second (0.80, 95% CI: 0.75–0.84) and third dimension (0.82, 95% CI: 0.78–0.86). In addition, the total reliability of the P-Vac-Cov19-DENT scale was calculated (0.89, 95% CI: 0.86–0.91) obtaining optimal results. Finally, each dimension was nominated according to the content of grouped items (Table 8).

**Table 8:** Final preparation of P-Vac-Cov19-DENT scale according to its dimensions

Dimensions	Items
**Perception about the organization**	**Q1.** I considered that the dissemination of those selected in first instance by the professional association and the call for the COVID-19 vaccination process were fast and efficient.
	**Q2.** I consider that the organization in relation to vaccination environment (adequate space, ventilation, signage) was the most adequate.
	**Q3.** I consider that the waiting period to start the vaccination process was justified for the benefit it represents.
	**Q6.** I believe I would recommend the COVID-19 vaccination service to my colleagues.
	**Q7.** I consider that the process of data verification and elaboration of the list order for vaccination was adequate and impartial.
	**Q8.** I consider that the geographical location of the vaccination process was the most appropriate
	**Q9.** I consider that the schedule chosen for the vaccination process was the most suitable one.
	**Q10.** I consider that the management of the Ministry of Health and the professional association to obtain vaccines was the best.
**Perception about care received**	**Q4.** I consider that the triage staff treated me with kindness and respect.
	**Q5.** I consider that the information received regarding the possible post-vaccine effects was sufficient and adequate.
	**Q11.** I consider that the treatment received by the health care personnel during the vaccination process was ideal.
	**Q13i.** I feel that at the time I was injected with the vaccine, the procedure was not transparent since I was not allowed to see what was being injected.
	**Q14.** I have constant concerns that my vaccine dose has been manipulated in some way
	**Q15i.** I am suspicious about the professional competence of the personnel involved in the care work of the COVID-19 vaccination process.
**Perception about vaccine efficacy**	**Q12.** I believe that the vaccine I received will prevent serious health complications if I become infected with the coronavirus.
	**Q16.** I am confident in the efficacy of the COVID-19 vaccine.
	**Q17.** I feel that being vaccinated against COVID-19 gives me the confidence to treat a larger number of patients.
	**Q18.** Now that I have been vaccinated against COVID-19 I am less afraid of getting infected with the coronavirus.

i: These items (Q13 and Q15) should be rated on an inverted Likert scale from 1 (strongly agree) to 5 (strongly disagree)

## Discussion

In the last 2 years, due to COVID-19, there has been a worldwide crisis in all areas, affecting the mental health and work environment of all citizens [22–25]. The vaccine is considered a promising solution to restore normality. Its scope and acceptance depend on the organization of the immunization process at governmental level, which should include the dissemination of reliable information on efficacy of the vaccine, the provision of adequate health care services and the availability of trained personnel.

Currently, there is no psychometric instrument that that measures perception of the vaccination process in health professionals since those available in literature are limited exclusively to evaluation of vaccine acceptance [6, 11–17]. Therefore, implications and consequences of the lack of comprehensive assessment of this process are unknown, and making these effects invisible could negatively affect the scope of immunization.

Due to the current epidemiological situation, dentists are at high risk of exposure to the COVID-19 virus when performing specific procedures that generate contaminated bioaerosols [3–5]. In addition, dentistry can be considered a stressful profession due to the legal problems that can arise from human error, the interrelationship with patients, routine work, compliance with regulations, and the risk of care, among others [26, 27]. In addition, the psychological impact of COVID-19 pandemic may have increased stress levels in dentists [27]. In view of the above, it is imperative to create a scale to validate how these professionals perceive the vaccination process, since it is essential to encourage their greater predisposition to be vaccinated, as their immunization increases protection against cross-infection by SARS-CoV-2 variants [28]. Furthermore, as health professionals, they are responsible for promoting best health practices, since their example is fundamental to generate public awareness and motivation to accept the vaccine.27–29 [29–31].

The P-Vac-Cov19-DENT items were constructed based on the literature and key words collected by previously conducting an in-depth interview with fifty dentists. This allowed us to recognize that excessive concern about becoming infected can produce different thoughts and behaviors in health professionals during the pandemic context, which could have an impact on public health [32]. It should be noted that the content of the scale was considered good when it was evaluated under nine aspects by five expert judges. On the other hand, by observing differences in correlations between items of the questionnaire, it is possible to review and address aspects of methodological structure of this strategy from a different contextual perspective [33]. However, thanks to the total item correlation, it was possible to identify items 13 and 15 as negative items, which made it possible to correct the direction of the score on the Likert scale. In addition, it may seem contradictory that item Q14 (I have constant concerns that my vaccine dose has been manipulated in some ways) did not show a negative correlation. This was probably due to reports in the press, including the broadcasting of a video, of some health personnel tampering with the vaccines by altering their dosage or administering them without any content in the syringe [34]. For all these reasons, item Q14 could have caused the dentists to agree or totally agree (4 or 5 points, respectively) with it. However, despite this concern, dentists went to get vaccinated en masse, which is understandable since it was a requirement for practicing dentistry. Many dentists also felt that they had no other choice since it was the only thing that existed at that time to deal with COVID-19. At the same time, it should be noted that due to the complaints, the authorities in charge of the vaccination process allowed, for the peace of mind of the subject to be vaccinated, that he/she or a companion could verify the dose administered, even allowing filming the moment of vaccination. For all of the above, it is also reasonable and concordant that the majority of dentists would disagree with items Q13 and Q15, so the answers had to be scored inversely according to what was observed in the correlation matrix.

Regarding the exploratory factor analysis and the sum of squared loadings extraction method, it was observed that all items were saturated in a four-dimensional structural model. However, according to the Parallel Analysis based on minimum rank factor analysis, three dimensions were obtained and this was justified by good sample adequacy indices. It should be noted that this analysis is currently the most widely accepted factor extraction method by the scientific community [35, 36]. It should be noted that in the early stage of the present study, a four-dimensional P-Vac-Cov19-DENT scale was designed to assess the “Perception about the organization” (Q1, Q2, Q3, Q7, Q8, Q9 and Q10), the “Perception about vaccine efficacy” (Q12, Q16, Q17 and Q18), the “General perception of the service” (Q4, Q5, Q6 and Q11) and the “Perception of the care procedure” (Q13, Q14 and Q15). However, according to the parallel analysis, the eighteen items of the P-Vac-Cov19-DENT scale were saturated in three factors that explained 62.82% of the total variance of the analysis, so it was decided to restructure the scale. For this reason, item Q6 was moved to the dimension “Perception about the organization”, while the dimension “Perception about vaccine efficacy” was maintained with the same items. Likewise, the two dimensions “General perception of the service” and “Perception of the care procedure” were merged into a single dimension that grouped together items Q4, Q5, Q11, Q13i, Q14 and Q15i, calling it the dimension “Perception about care received” given its content. On the other hand, the presence of the 18 items was supported by principal component analysis with varimax rotation, but the items had to be reordered according to the dimensions. Likewise, to guarantee the reliability of the internal consistency for each dimension and the total scale, Cronbach’s alpha was calculated, yielding optimal results.

The items considered in this P-Vac-Cov19-DENT scale addressed the dimension of perception about the organization, which included efficiency of the means of dissemination for vaccination process, impartiality for the selection of beneficiaries, coordination with the competent authority, adequate arrangement of the environments and their geographical location, suitability of the vaccination schedule and waiting time for the beginning of the process. These were considered in order to obtain the necessary information for governments to make decisions with the objective of improving the development of this process and facilitating access to all people to be vaccinated, avoiding unpleasant, uncomfortable or painful situations that would lead to low turnout in the next vaccinations [37–39]. In addition, the scale proposed in the present study took into account the perception of vaccine efficacy, which considered the protection generated by the vaccine and the possibility of resuming health care work with minimal risk of contagion and complications. This is important to consider since adequate information regarding the vaccine offered must have appropriate scientific support and use appropriate channels of dissemination to prevent the user from feeling uncertainty or rejection of the immunization [9, 39]. The perception of the care received was also considered as a dimension, which included the empathy of the health care personnel, adequate information regarding adverse effects and the possibility of recommending the service. It is crucial to include in the vaccination process friendly health care personnel with adequate bidirectional communication to ensure the beneficiary’s peace of mind, also taking into account the transparency of the procedure and trust in the health care personnel. The vaccination process must have adequately trained health employees to ensure that the management is carried out with clarity, credibility and security [9, 39].

Success in the immunization process guarantees the eradication and control of diseases and is key to public health [40, 41]. Furthermore, considering that the decision to be vaccinated depends to a large extent on prior knowledge, experience and beliefs [42], this scale would help the competent authorities to obtain more information on the needs and expectations that dentists have about the COVID-19 vaccine, since these professionals are exposed to treating patients potentially infected by the COVID-19 virus because of the work they perform [43]. However, it should be recognized that the way in which the scale items have been written could be applied not only to dentists but to any health professional who performs health care work.

The importance of this P-Vac-Cov19-DENT scale is that it could allow government authorities to expand their knowledge about the vaccination process to generate changes in immunization readiness and improve the reach of immunization, which would help ensure its sustainability and accessibility over time for future requirements.

Among the limitations of the present study, it was not possible to perform a criterion analysis of this scale by external validation, because no scale measuring the perception of the vaccination process in dental professionals was found in the current available literature (February 2022), so the results obtained in this preliminary validation should be taken with caution. Therefore, testing convergent and discriminant validity is recommended as a future direction. On the other hand, it was not possible to interview the participants in person, because at the time the study was carried out the Peruvians were in mandatory social isolation [23]. We can also mention as a limitation that the present study was carried out only in a population of dental professionals working in two universities in the Peruvian capital. In addition, an analysis of instrument stability was not carried out. For this reason, we recommend testing and retesting this scale at two different times and evaluating the concordance of the scores [44]. However, the P-Vac-Cov19-DENT can be applied in several countries and in different health professions that perform health care work, since it has demonstrated good internal consistency. Therefore, it is also recommended to adapt this scale to other social realities and to future immunizations of highly contagious diseases, as this will make possible to recognize the shortcomings and strengths of the vaccination process, and thus make these results known to the competent authorities in order to improve planning and management of the process.

## Conclusion

In conclusion, acknowledging the limitations of the present study, the scale for the perception of COVID-19 vaccination process in dental professionals proved preliminarily to be valid and reliable and can be used for research purposes. However, it is recommended to extend its application and evaluate its metric properties in other health professionals.

## Data Availability

Data are available upon request at cesar.cayo@upsjb.edu.pe

## Abbreviations

COVID-19: Coronavirus disease 2019
CI: Confidence interval
KMO: Kaiser-Meyer-Olkin
P-Vac-Cov19-DENT: Perception scale of the COVID-19 vaccination process in dental professionals
SARS-CoV-2: Severe acute respiratory syndrome caused by coronavirus 2
STROBE: Strengthening the Reporting of OBservational studies in Epidemiology
SPSS: Statistical Package for the Social Sciences
